# How Medical Representatives Influence Prescribers Behavior? 

**Published:** 2012

**Authors:** Fanak Fahimi

*It’s my job to figure out what a physician’s price is. For some it is dinner at the finest restaurants, for others it is enough convincing data to let them prescribe confidently and for others it is my attention and friendship...but at the most basic level, everything is for sale and everything is an exchange.*

*Shahram Ahari*

The statement above is only a quotation and I am not debating if it is true or false. I leave it for the readers to think and judge. 

In any setting, the process of selling involves getting in touch with potential customers, recognizing their requests, convincing them that your products or services are more pleasant than those of competitors and can best please the customer.

Pharmaceutical marketing is a specialized field where medical representatives form its skeleton. In 2000, drug companies spent more than 15.7 billion dollars on promoting prescription drugs in the United States. Medical representatives aim to influence prescription pattern of doctors in favor of their brands. Medical representatives (“medreps” or “drug reps” or “reps”) act by arranging appointments with prescribers, pharmacists and other hospital medical teams.

Drug reps increase drug sales by influencing physicians, and they do so with finely titrated doses of friendship. They sometimes deliver lectures in medical work places or at a hotel or conference venue.

They are recognized as key people who can sponsor conferences for physicians and arrange for travel expenses.They also deal with budgets for other purposes such as catering, outside speakers, conferences, hospitality, *etc*.

Normally, medical sales executives have their own regional area of responsibility and plan how and when to target health professions. Medreps should be able to keep up with the latest clinical data supplied by the company and interpreting, presenting and discussing these data with health care providers during their periodic visits.

They should also maintain update knowledge about competitor products and articles published regarding them.

Being smart, dedicated, good psychologist, well groomed, having soft skills with good personality and a high Emotional Quotient (EQ), would be part of their success towards their performance.

Factors contributing to a drug selection are comprised of product quality, safety, effectiveness, price, availability, company reputation, packaging attraction, patient affordability and satisfaction. 

It has been shown that a sharp drop in price by the competitor brand is an important reason for a physician to shift his/her prescription to the competitor brand.

One should not ignore the influence of regular visit of medreps, samples given at visits, sponsorship for conferences, personal gifts, and other incentives and promotional tools in selecting a medication for a patient.

Considering the impact of the mention dimensions on the physicians, profiles, it is possible to understand the ‘why’ behind their prescribing behavior.

This is not to say that profit is evil or that business is evil. The pharmaceutical industry has done some great work, developing several drugs in the past years. They have been a blessing for many people, who in many cases, save money by avoiding costlier, or more invasive treatments.

*Fanak Fahimi* *is currently working as an Associate Professor at the Department of Clinical Pharmacy, School of Pharmacy and Chronic Respiratory Disease Research Center (CRDRC), NRITLD, Masih Daneshvari Hospital, Shahid Beheshti University of Medical Sciences Tehran, Iran. She could be reached at the following e-mail address:* *fanakfahimi@yahoo.com*

